# Egg Yolk Oil as a Plasticizer for Polylactic Acid Films

**DOI:** 10.3390/membranes12010046

**Published:** 2021-12-29

**Authors:** María Carpintero, Ismael Marcet, Manuel Rendueles, Mario Díaz

**Affiliations:** Department of Chemical and Environmental Engineering, University of Oviedo, C/Julian Clavería 8, 33006 Oviedo, Spain; carpinteromaria@uniovi.es (M.C.); marcetismael@uniovi.es (I.M.); mariodiaz@uniovi.es (M.D.)

**Keywords:** egg yolk oil, polylactic acid, plasticizer, food packaging, photoprotective

## Abstract

Polylactic acid (PLA) is known to be one of the most extensively used biodegradable thermoplastic polyesters, with the potential to replace conventional petroleum-based packaging materials; however, the low flexibility of films prepared using PLA has limited the applications of this biopolymer. In this study, in order to improve the mechanical properties of PLA films and to provide them with antioxidant properties, egg yolk oil was used as a biobased plasticizer. For this purpose, PLA films with increasing concentrations of egg yolk oil were prepared and the effects of this oil on the light transmission, transparency, colour, water vapour permeability, solubility, antioxidant activity and mechanical properties of the films were characterized. In addition, electron microscopy of the structure of the transverse section of the films was also performed. Results showed that the formulations with higher concentrations of egg yolk oil increased the films’ elasticity, and their light barrier and antioxidant properties. Finally, in order to test the films as a packaging material for food applications, extra virgin olive oil and resveratrol, both photosensitive compounds, were packed and exposed to ambient light. Overall, the results show the potential of egg yolk oil as an environmentally friendly plasticizer that can improve the flexibility of PLA films and provide them with additional photoprotective properties.

## 1. Introduction

Polylactic acid (PLA) has been reported as one of the most promising biopolymers for the preparation of bioplastics capable of substituting petrol-based plastics [1]. PLA belongs to the family of aliphatic esters, being the biodegradable polymer of lactic acid, which is produced as an intermediate or by-product of animal and plant metabolism [2]. Although in the first half of the twentieth century the production of PLA was expensive and its use was limited to medical applications, nowadays it is relatively cheap, and it is expected that the production of this polymer will reach 30 thousand tons by 2024 [1]. In addition to being prepared from renewable sources, it must be highlighted that its natural decomposition produces water and carbon dioxide, it is biocompatible, and it can be moulded in various shapes. However, this biopolymer also has certain mechanical limitations such as poor flexibility, which can be improved by incorporating plasticizers in the film-forming solution or modifying the PLA surface [3]. 

There are several types of plasticizers, and among them, petroleum phthalate plasticizers are the most demanded and produced internationally, although their use is being restricted because of threats to human health and the environment [4,5]. In this context, the development of non-toxic and biodegradable plasticizers is a topic that is attracting researchers’ attention in recent years. In the case of PLA, biobased plasticizers like polyethylene glycol, citrate esters, paraffins, triacetin and oligomeric lactic acid have been used to improve the flexibility and ductility of PLA, but at the cost of stiffness and tensile strength, which often deteriorate with the addition of these compounds [6,7]. With respect to the question of plasticizers, a novel and promising additive that could be used for this purpose in PLA is egg yolk oil. Egg yolk oil has been widely used as an ingredient in human nutrition, where it is of interest due to its fatty acid profile and high content of soluble vitamins and lecithin. Egg yolk oil has percentages of oleic acid and palmitic acid of 52.61% and 22.72%, respectively [8], which are close to those of the fatty acids found in vegetable oils, such as olive oil [9]. In this sense, many authors have reported on the use of vegetable oils and fatty acids, such as epoxidate olive oil, coconut oil, cottonseed oil, palm oil, soybean oil and palmitic, stearic and oleic acids as plasticizers for PLA-based materials [10,11,12,13,14]. In addition, egg yolk oil shows a high concentration of carotenoids, which are bioactive compounds with antimicrobial, photoprotective and antioxidant properties, which could allow the use of the egg yolk oil not only as a plasticizer for PLA-based bioplastics, but also as a bioactive compound that provides extended properties to the materials in which it is incorporated [15]. Furthermore, increasing the range of applications for the oily fraction of egg yolk makes the separation of egg yolk compounds into the proteinaceous and the lipidic fraction more commercially feasible, which would allow their specific utilization in different food systems and hence the revalorization of the whole egg yolk. One such application is the use of the delipidated protein fraction to prepare packaging materials [16].

Therefore, the aim of this research is to study the performance of egg yolk oil not only as a plasticizer but also as an additive with bioactive properties for PLA-based materials. The effect of the incorporation of this egg yolk oil on the film’s mechanical and antioxidant properties was tested. Finally, in order to evaluate the antioxidant and light barrier properties of the prepared materials in a real-case scenario, resveratrol solutions and extra virgin olive oil were introduced into small bags made with the PLA films containing egg yolk oil.

## 2. Materials and Methods

### 2.1. Egg Yolk Oil: Extraction and Carotenoids Quantification

Eggs were acquired in a local market and their yolk and white were manually separated. The egg yolk surface was carefully dried with blotting paper, the vitelline membrane was punctured with a pair of tweezers and the egg yolk was recovered in a beaker and lyophilized (Telstar, Cryodos-80) while the vitelline membrane was discarded. For the egg yolk oil extraction, a mixture of two organic solvents with different polarities, 2-propanol and hexane, in a 3:7 ratio was used [8].

Afterwards, the lyophilized egg yolk was mixed with this solution at a ratio of 1:10 (*w*/*v*). The extraction was carried out by magnetic stirring for 30 min at room temperature and then the mixture was vacuum filtered to remove the precipitate. The organic solvents in the permeate fraction were recovered with a Büchi R-205 rotary evaporator (Büchi Labortechnik AG, Flawil, Switzerland) at 60 °C. The recovered egg yolk oil showed a deep orange colour and a viscous appearance. 

The determination of the amount of carotenoids in the egg yolk oil was performed according to the standard method UNE-EN ISO 17932:2012 [17]. Briefly, between 100 and 500 mg of yolk oil were weighed in a 25 mL volumetric flask and made up to the mark with isooctane. After shaking vigorously, the solution absorbance was measured at 446 nm and the total carotenoid content was determined in mg per kg of sample using the Equation (1):

(1) $$Wc=\left(10^{6}\times A\right)/(2610\times l\times \rho )$$

where 2610 ($g\times 100{\;\mathrm{mL}}^{-1}\times {\mathrm{cm}}^{-1}$) is the specific extinction coefficient of a 1% solution of β-carotenes in isooctane at 446 nm; *A*, the absorbance of the sample at 446 nm; *l*, the length of the cell (1 cm); and ρ, the concentration of the sample in grams per 100 mL.

### 2.2. Preparation of PLA Films with Egg Yolk Oil

The film-forming solution was prepared by dissolving PLA (4031D; NatureWorks, Minnetonka, MN, USA) in dichloromethane (5%, *w*/*v*), with continuous magnetic stirring at room temperature. Subsequently, different volumes of egg yolk oil stock were added and gently stirred for 5 min, obtaining final concentrations of 0.1, 0.2 and 0.3 g of oil per gram of PLA (film A1, A2 and A3, respectively). Finally, 5 mL of every film-forming solution was poured into a glass plate, such that 0.1 mL of film-forming solution was cast per cm^2^ of the glass plate surface. 

Films were dried at room temperature for 15 min, and then peeled off the plate and stabilized in a humidity chamber (HCP50; Memmert, Schwabach, Germany) at 25 °C and 50% humidity for 2 days.

### 2.3. Physical Properties of PLA Films Loaded with Increasing Concentrations of Egg Yolk Oil

#### 2.3.1. Light Transmission and Transparency

The barrier properties of films against ultraviolet and visible light were assessed according to the methodology followed by Dick et al. [18]. For this purpose, films were cut into rectangular pieces of 1 cm width and placed inside a glass spectrophotometer cell. The light transmission of the samples was tested using a UV-Visible Genesys 150 spectrophotometer (Thermo Scientific, Waltham, MA, USA), from 280 to 800 nm, using an empty test cell as a blank. The transparency of the films was calculated according to the Equation (2):

Transparency = *A*_600_*/x* (2)

where *A*_600_ is the absorbance of the film sample at 600 nm and *x* is the film thickness (mm).

Film thickness was measured with a micrometer (Mitutoyo, Kanagawa, Japan), with a precision of ±1 µm. The thickness was measured in seven different areas, one of them in the centre of the film and the other six around the film perimeter.

#### 2.3.2. Colorimetric Properties

Film colour and colorimetric properties were measured using a LC 100/SV 100 spectral colorimeter (Lovibond, Amesbury, UK) to assess the L* value (lightness), a* value (redness/greenness), and b* value (yellowness/blueness) of the films. 

To carry out the analysis, the films were placed on a white sheet (L*, 98.3; a*, −0.9; b*, 0.9), which was used as a white standard. The total colour difference *(*∆E*) was calculated using Equation (3), where ∆L*, ∆a* and ∆b* are the difference values of the corresponding colour parameters between the films with egg yolk oil and the control film without egg yolk oil. The whiteness index (*WI*) and the chroma of each film were calculated with Equations (4) and (5), respectively [19,20]. Each determination was carried out in triplicate.

(3) $$\Delta E^{*}=\sqrt{{\left(\Delta L^{*}\right)}^{2}+{\left(\Delta a^{*}\right)}^{2}+{\left(\Delta b^{*}\right)}^{2}}$$

(4) $$WI=100-\sqrt{{\left(100-L\right)}^{2}+a^{2}+b^{2}}$$

(5) $$Chroma=\sqrt{a^{2}+b^{2}}\;\;\;\;$$

#### 2.3.3. Mechanical Properties

The mechanical properties of the PLA films with different concentrations of egg yolk oil were analysed with a puncture test using a TA.XTPlus Texture Analyser (Stable Microsystems, Godalming, UK), equipped with a 5 kg load cell and a 5 mm diameter probe (P/5S). 

Films were cut into four square pieces of equal size (2 × 2 cm) and placed in the texturometer between two plates, making sure that the film was well stretched and correctly in place, without wrinkles. The plates used to attach the film sample have a hole that allows contact of the film with the probe, which presses until the film breaks. In this case, the speed of the probe was 1 mm/s and the contact force 1 g. The puncture resistance (*PS*) and puncture deformation (*PD*) values were calculated using Equations (6) and (7), respectively:

*PS = Fm/Th* (6)

(7) $$PD=\left(\sqrt{D^{2}+R^{2}}-R\right)/R$$

where *Fm* (N/mm^2^) is the maximum force applied by the probe before the film breaks, *Th* is the thickness of the film, *D* is the distance covered by the probe from the time it makes contact with the film until it breaks, and *R* is the radius of the film breakage hole [21].

#### 2.3.4. Water Vapour Permeability and Solubility

The water vapour permeability of the films was tested according to the methodology used by Weng et al. [21]. Briefly, polyvinyl chloride cups were filled with distilled water and sealed with circular pieces of the films cut with the same diameter as the cup mouth, taking care that no gaps remained between the cup and the piece of film, which had to remain intact. A height of 1 cm was left between the water surface and the PLA films. Finally, the cups, filled with water and sealed, were weighed and placed in a silica gel desiccator, and the change in their weights was register red every hour over a 7 h period. The weight loss was plotted versus time and the water vapour transmission rate (*WVTR*) was calculated according to Equation (8):

*WVTR* = *G*/(*t* × *A*) (8)

where *G*/*t* is the change in the cup weight per unit of time (g/h), the slope of the graphical representation, and *A* (m^2^) is the area of the cup mouth covered by the film.

The WVTR values were used to calculate the water vapour permeability (*WVP*) of the films by means of Equation (9):

*WVP* = (*WVTR* × *Th*)/∆*P* (9)

where *Th* (mm) is the thickness of the film and ∆*P* (kPa) is the difference in partial vapour pressure between both sides of the film.

The solubility measurement was also performed according to Weng et al. [21]. Briefly, PLA films with egg yolk oil were cut into 2 cm diameter circles and dried in an oven at 90 °C for 24 h to determine their dry weight. Other intact film fragments were immersed in a 0.1 M Trizma buffer solution pH 7.0 at room temperature for 24 h. Undissolved film remains were recovered by vacuum filtration, dried at 90 °C for 24 h and weighed. The percentage of dissolved film was calculated with the following Equation (10):

*S* (%) = (*m*1 − *m*2)/*m*1 × 100 (10)

where *S* (%) is the percentage of solubilised film, *m*1 is the initial dry weight of the film and *m*2 is the dry weight of the undissolved film remains in grams.

#### 2.3.5. Scanning Electron Microscopy (SEM)

A JSM-6610LV scanning electron microscope (JEOL, Peabody, MA, USA) was used to study the microstructure of the transverse section of the PLA films with egg yolk oil. For that purpose, film samples were cut into square pieces of 1 × 1 cm using a surgical blade. Film fragments were attached to metal bases with a double-sided adhesive carbon strip, so that one side of the strip was attached to the support and the other side to the film fragment, and then the films were gold-sputter-coated for 5 min under an argon atmosphere. The micrographs were taken at magnifications between 2000× and 3000×, and the voltage was set at 20 kV.

#### 2.3.6. Antioxidant Activity

The free radical scavenging activity of the egg yolk oil incorporated in the PLA films was analysed using the DPPH free radical method. For that purpose, a stock solution of DPPH in isopropanol-hexane (30:70) was prepared by mixing 16 mg of DPPH (1805; Cayman Chemical Company, Ann Arbor, MI, USA) with 40 mL of the solvent mixture. Films were then cut into 1 × 2 cm strips and placed in glass jars, to which 15 mL of isopropanol-hexane and 1 mL of the prepared DPPH stock solution were added. The bottles were stored in darkness and the liquid absorbance at 517 nm was measured every 24 h, using an isopropanol-hexane solution as the blank. After each measurement, the liquid extracted for the absorbance measurement was returned to jars. The antioxidant activity of films was calculated by the following Equation (11):

Antioxidant capacity (%) = (*Abs_c_* − *Abs_s_*)/*Abs_c_* × 100 (11)

where *Abs_c_* is the absorbance at 517 nm of the control, PLA film without egg yolk oil; and *Abs_s_* is the absorbance at 517 nm of the sample, PLA films with different concentrations of egg yolk oil [20].

### 2.4. Film Application as Active Packaging

#### 2.4.1. Film Application as Active Pouches to Pack a Resveratrol Solution

A resveratrol stock solution was prepared by dissolving 15 mg of resveratrol (R0071; TCI, Tokyo, Japan) in 500 mL of distilled water; the mixture was left in agitation for 24 h, completely protected from sunlight, to achieve the total dissolution of resveratrol. 

To determine whether the barrier properties of films were enough to protect the resveratrol solution from sunlight, the same film pouches that were subsequently used to contain the olive oil were prepared to contain 4 mL of the resveratrol stock solution. Pouches were exposed to photo-oxidizing conditions, and the increase in the concentration of the cis form of the resveratrol, which indicates its inactivation, was measured every 24 h for 2 days, examining the absorbance of the packed resveratrol solutions at 304 and 286 nm [22]. In addition, a glass dish containing 10 mL of free resveratrol solution, subjected to the same oxidative conditions, was used as a positive control. To calculate the concentrations of cis-resveratrol and trans-resveratrol in the solutions and their variation over time, Equations (12) and (13) were used:

(12) $$C_{trans}=\frac{A_{2}-\left(\varepsilon_{2cis}/\varepsilon_{1cis}\right)\cdot A_{1}}{l\cdot (\varepsilon_{2trans}-\left(\varepsilon_{2cis}/\varepsilon_{1cis}\right)\cdot \varepsilon_{1trans}}$$

(13) $$C_{cis}=\frac{A_{2}-\left(\varepsilon_{2trans}/\varepsilon_{1trans}\right)\cdot A_{1}}{l\cdot (\varepsilon_{2cis}-\left(\varepsilon_{2trans}/\varepsilon_{1trans}\right)\cdot \varepsilon_{1cis}}$$

where *A*_1_ and *A*_2_ are the absorbance of the resveratrol solution at 304 and 286 nm, respectively; *ε*_1*trans*_ and *ε*_2*trans*_ are the molar extinction coefficients of trans-resveratrol at 304 nm and 286 nm, respectively; *ε*_1*cis*_ and *ε*_2*cis*_ are the molar extinction coefficients of cis-resveratrol at 304 nm and 286 nm, respectively; and *l*, the length of the cell (1 cm). The molar extinction coefficients of the trans-resveratrol and cis-resveratrol are 30,335 and 9515 M^−1^ cm^−1^ for a wavelength of 304 nm and 23,400 and 14,986 M^−1^ cm^−1^ for 286 nm, respectively [22].

#### 2.4.2. Film Application as Active Pouches to Pack Extra Virgin Olive Oil

Film pouches were made in order to pack olive oil as a food product in individual portions. Briefly, 7 mL of olive oil was incorporated in the pouch that showed the highest antioxidant properties according to the procedure described above, using as the control a bag prepared with PLA films alone. Containers were prepared with a conventional plastic sealer by heating and sealing the PLA films in such a way that the pouches had a rectangular shape. The olive oil samples contained in the PLA film pouches were subjected to oxidizing conditions for 5 days by keeping them directly exposed to ambient light, according to the procedure described by [23]. As the positive control, 7 mL of extra virgin olive oil in an open petri dish was subjected to the same oxidative conditions. To determine the progress of the olive oil samples’ oxidation, changes in the concentration of peroxides over 3 days were measured by calculating the peroxide value (PV) for each sample every 24 h.

The PV of samples was determined according to the standard method ISO 3976 | IDF 74 [24]. Briefly, 15 mg of oil was weighed and dissolved in 9.8 mL of a 7:3 (*v*/*v*) chloroform-methanol mixture; subsequently, 50 µL of ammonium thiocyanate and 50 µL of Fe(II) solution was added. Samples were shaken and kept in the dark for 10 min, and then the absorbance was measured at 500 nm against a blank. The blank contained all the previous reagents except the oil samples. The PV, expressed as mEq O_2_/Kg oil, was calculated with the following formula:

(14) $$\mathrm{PV}=\frac{Abs}{55.84\times w}\times \frac{1}{b}\;[{\mathrm{mEq}\;O}_{2}/\mathrm{Kg}\;\mathrm{oil}]$$

where *w* is the weight of oil in g, *Abs* is the absorbance of the sample, 55.84 is the atomic weight of Fe(III) and *b* is the slope of the Fe(III) calibration curve. 

For the calibration curve, a 1 mg/mL solution of Fe(III) chloride in distilled water was prepared, from which a 10 µg/mL Fe(III) stock solution in chloroform/methanol was made. From the 10 µg/mL stock solution, standards containing 5, 10, 20, 30, 40 and 50 µg Fe(III) were prepared and made up to 9.8 mL with chloroform-methanol (7:3). Subsequently, 50 µL ammonium thiocyanate was added and the absorbance was measured at 500 nm. The absorbance of the Fe(III) standards was plotted versus their concentrations.

### 2.5. Statistical Analysis

Each of the different tests was carried out in duplicate, and the average and the corresponding standard deviation of the results obtained were represented in each case. A simple analysis of variance (ANOVA), with a confidence level of 95%, was performed to determine the significant differences between the tested samples. The analysis was performed using Statgraphics Centurion XVI.

## 3. Results and Discussion

### 3.1. Egg Yolk Oil

As a result of the extraction of egg yolk oil with organic solvents, a viscous orange solution was obtained. This oily solution contained a concentration of 60 µg of carotenoids per g of yolk oil, which was in agreement with other studies carried out to date [8,25,26]. In addition to its potential use as a PLA plasticizer, the presence of these bioactive compounds in the egg yolk oil makes it an additive of interest for creating active food packaging with possible antioxidant properties.

### 3.2. Physical Properties of PLA Films Loaded with Increasing Concentrations of Egg Yolk Oil

#### 3.2.1. Visual Aspect, Light Transmission and Transparency

**Figure 1:**
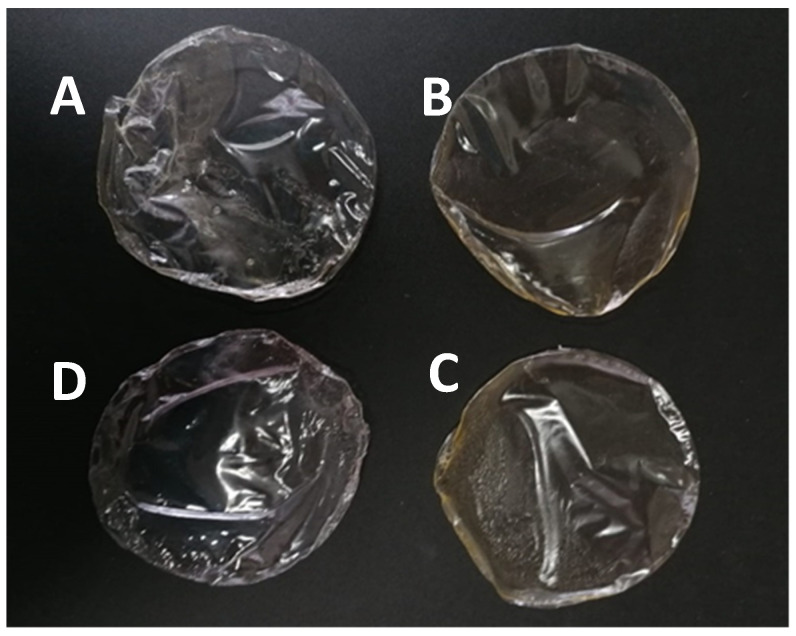
Visual aspect of PLA films with egg yolk oil concentrations of 0.1 g per g of PLA (A1, (**A**)), 0.2 g/g PLA (A2, (**B**)) and 0.3 g/g PLA (A3, (**C**)); and of the control PLA film (**D**).

Films with increasing concentrations of egg yolk oil were easily peeled from the glass dishes in one piece, with those with higher concentrations of oil being easier to peel than those containing less oil. Films with egg yolk oil were homogeneous, significantly more opaque than the standard PLA films and with a slight orange coloration (Figure 1).

Light transmission properties of the films were evaluated at wavelengths from 200 to 800 nm and light transmittance values were recorded (Table 1). Sunlight is a potent oxidizing agent for lipids and other photosensitive compounds present in food, cosmetics and other materials, which is why low transmittance values are essential for barrier materials. Films made with PLA and egg yolk oil have lower UV/Vis light transmission percentages than normal PLA films; this suggests that egg yolk oil endows the PLA films with the ability to act as a barrier against ultraviolet light (200–300 nm) and visible light (300–800 nm). This property of the films with egg yolk oil extract is likely due to the presence of carotenoids, which have been shown to act as a barrier against UV/Vis light [27]. As with transmittance, the addition of egg yolk oil also affected the transparency of films, which decreased as the amount of added oil extract increased (Table 1), augmenting their barrier properties as predicted above.

#### 3.2.2. Colorimetric Properties

The colour attributes of the PLA films containing egg yolk oil are shown in Table 2. It can be observed that when egg yolk oil was incorporated into PLA films, the b* values increased, which means that the prepared materials acquired a yellowish coloration; however, the addition of oil did not have any noticeable effect on the lightness or redness of the films. This could be explained by the fact that the main pigments found in egg yolk oil are carotenoids, and in particular β-carotene, which has a yellow-orange colour and hence exerts more influence on the b* than the a* coordinate.

In the case of the total colour difference (∆E*) between the films with egg yolk oil (A1, A2, A3) and the PLA film (control), it can be observed that the values for this parameter were higher than 3.0 for the A2 and A3 films, which indicates that the colour changes produced in the material due to the addition of egg yolk oil would be appreciated by the human eye. These ∆E* results agree with chroma values, the total amount of colour, which increase with pigment concentrations, and explains why films with higher concentrations of egg yolk oil have a yellowish-orangish coloration. Conversely, the WI values decrease with increasing amounts of added oil, and this is because of the presence of chromophore groups in the egg yolk oil carotenoids, which decrease the film whiteness [20].

#### 3.2.3. Mechanical Properties

The addition of egg yolk oil to PLA films had a noticeable effect on its mechanical properties (Table 3). In terms of film thickness, a tendency was detected for the value of this parameter to increase as the amount of egg yolk oil added to the PLA films increased. Despite these being very small changes, there were statistically significant differences between the control film and the films with the highest concentration of egg yolk oil, as might be expected considering the amount of oil that was introduced in the A2 and A3 film formulations. 

The addition of egg yolk oil also affected other mechanical parameters of these materials, such as their puncture deformation (PD) and their puncture strength (PS) (Table 3). Regarding the PD parameter, the plasticizer saturation occurs at some point in the range between 0.1 and 0.2 g of egg yolk oil per gram of PLA, since the A1 films were the most flexible films tested, showing an increase in their flexibility of 171% compared to the control films, but the A2 films showed a decrease in the PD value in relation to the A1 formulation. This decrease in the flexibility of the A2 films, that went even further for the A3 films, may be explained by an increase in the interactions between the plasticizer compounds, which could produce a phase separation phenomenon [28]. Overall, these results agree with those obtained by Stoll et al. [19] and Carbonell-Verdu et al. [11], who observed that adding small amounts of carotenoids and cottonseed oil to PLA films also increased their elasticity.

Regarding the PS parameter, which represents the amount of energy needed to break a film, this decreased as the amount of oil added to the films increased. This decrease in the PS values obtained occurred even at the lowest concentration of egg yolk oil tested, which could be because the plasticizers act by decreasing the number and strength of interactions between polymers, favouring the handling of the films prepared but decreasing their overall mechanical strength. These results support those obtained by other authors, who observed that adding vegetable oils to PLA-based materials also decreased their strength, even at the lowest concentration of these plasticizers tested [11,29]. 

These results show that the addition of egg yolk oil to the film-forming solution has a clear plasticizer effect, producing discontinuities in the film matrix that allow the use of the PLA films as packaging materials.

#### 3.2.4. Water Vapour Permeability (WVP) and Solubility

Some of the most important properties of the polymers used for food packaging are their permeability and action as a barrier to water vapour, gases and aroma molecules [30]. WVP, in particular, is an important parameter for food packaging applicability, since the prevention or reduction of moisture transfer between food and the environment is one of the main functions of such a polymer [31]. The WVP of biopolymer-based films depends on factors such as the type of plasticizer used and the amount of additives introduced in the film-forming solution, and is a property closely related to the films’ microstructure [21].

In these experiments, as expected, the films A1 and A2 showed a decrease in the value of the WVP parameter in relation to the control PLA film of 44.8 and 14.5%, respectively. As any other non-polar compound, egg yolk oil possesses a lipidic nature and, hence, has the ability to hinder the diffusion of water molecules through the film matrix. The same was observed by Yahyaoui et al. [32] when small concentrations of essential oils were added to PLA films; however, when the egg yolk oil concentration was increased to 0.3 g oil/g PLA (A3 films), a noticeable increase in the value of the WVP parameter in relation to the control films was observed (Table 3). This may be due to the increase in the concentration of lipid molecules in the film matrix disrupting the microstructure of the material, with the biopolymers forming a looser network, leading to an increase in the diffusion of water through the films.

The solubility values of the films tested show that the addition of different concentrations of egg yolk oil to PLA films did not affect their solubility, which remained around 0% in all cases. This agrees with the expected results, since the PLA is soluble only in organic solvents such as chloroform, acetonitrile or dichloromethane and, therefore, its solubility in polar solvents like water or alcohols is null or insignificant [30,33].

#### 3.2.5. Microstructure

**Figure 2:**
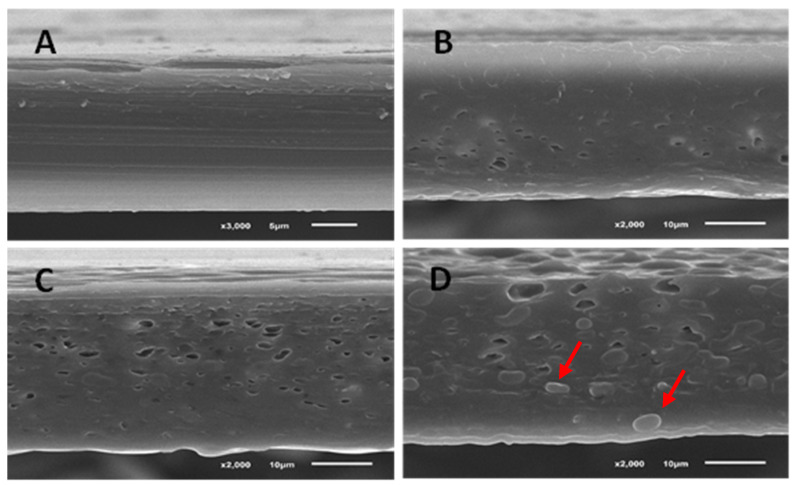
Micrographs of PLA films with egg yolk oil. (**A**) PLA film without oil. (**B**) PLA films prepared from the film-forming solution with egg yolk oil concentrations of 0.1 g per g of PLA (A1), (**C**) 0.2 g/g PLA (A2), (**D**) 0.3 g/g PLA (A3). In micrograph (**D**) the red arrows indicate two droplets of egg yolk oil in the PLA matrix (island-and-sea morphology).

The cross-sections of the PLA films with and without the incorporation of egg yolk oil are shown in Figure 2. As was expected according to the mechanical and permeability tests previously performed, the addition of oil had a clear effect on the internal structure of the films. Results show that the control PLA film presented a homogeneous, compact, continuous and smooth matrix, without pores or bubbles, and was perfectly structured; however, as the amount of oil added to the films was increased, an increase in the number of pores in the film matrix was observed. In the case of the film with the highest amount of egg yolk oil (A3, Figure 2D), in addition to pores, the film matrix presented granules or bubbles that reveal the existence of two phases or what is known as an island-and-sea morphology, in which the oil extract is dispersed in droplets, red arrows, in the PLA matrix [34]. These results agree with other studies performed, where it is shown that high oil concentrations used as PLA plasticizers produced a saturation effect in the film matrix, generating phase separation [35]. This island-and-sea morphology may be responsible for the higher WVP and poorer mechanical properties of the A3 film with respect to the rest of the materials tested, since these granules and pores in the PLA matrix may favour the diffusion of gases and explain the lower breaking strength and elasticity of these films.

#### 3.2.6. Antioxidant Activity

**Figure 3:**
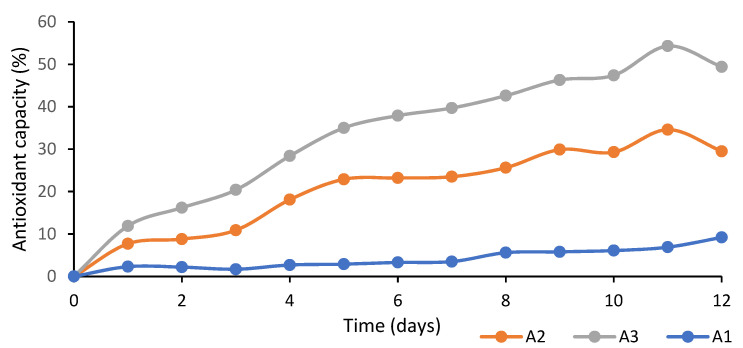
Changes in antioxidant capacity of PLA films with egg yolk oil concentrations of 0.1 g per g of PLA (A1), 0.2 g/g PLA (A2) and 0.3 g/g PLA (A3).

Figure 3 shows the antioxidant capacity of the PLA films loaded with several concentrations of egg yolk oil. As can be observed in this figure, the antioxidant activity of the films increased as the egg yolk oil concentration and experimental time increased. These films’ antioxidant capacity was due to the presence of carotenoids, such as lutein and zeaxanthin, in the egg yolk oil composition [36,37]. The antioxidant capacity of these carotenoids is mainly due to the presence of multiple conjugated double bonds in their molecular structure, which are able to easily react with oxygen free radicals, removing them from the reaction medium. In this sense, zeaxanthin shows a greater antioxidant and photoprotective capacity than lutein, since it has an additional double bond [38].

According to Figure 3, the antioxidant capacity of the tested films increased steadily until day nine, which suggests that the carotenoids retained in the PLA films were progressively released, after which a stagnation in the removal of DPPH free radicals from the media was observed, for which reason the experiment was concluded.

### 3.3. Film Application as Active Packaging

These experiments were carried out only with films loaded with the highest amount of egg yolk oil, the A3 films, since they showed the highest antioxidant and light barrier properties. Although their mechanical properties were poorer than those measured for films A1 and A2, they were good enough to easily prepare the proposed packaging materials.

#### 3.3.1. Film Application as Active Pouches to Pack Resveratrol

The films’ antioxidant and barrier capacities described before suggest that these materials could perform well in protecting food-related bioactive compounds from environmental light radiation. In order to study these protective effects, the isomerization of trans-resveratrol to cis-resveratrol was studied when sunlight was incident on the PLA bags loaded with a water solution containing trans-resveratrol, since this isomerization is highly light sensitive and involves a decrease in the bioavailability of this compound [39]. In addition, it must be pointed out that resveratrol can be found in a broad variety of vegetable sources [40], so it is a photosensitive bioactive molecule commonly present in foodstuffs. 

The results obtained are shown in Table 4, starting from an initial 100% trans-resveratrol solution. It can be observed that after 24 h of exposure to sunlight, the positive control, the free, unenclosed resveratrol solution, reached 88.25% isomerization (cis-resveratrol), and the resveratrol solutions protected by films reached 85.29 and 77.70% isomerization for the PLA film and the PLA film loaded with egg yolk oil, respectively. Therefore, it was observed that the PLA itself had a barrier effect against environmental light radiation, but the addition of egg yolk oil increased the photoprotective effect of the materials prepared, achieving up to 10.55% of additional protection in the first 24 h. In order to preserve the resveratrol bioactivity from the light radiation, other researchers have encapsulated it in niosomes or in microparticles, obtaining low levels of isomerization when these particles were irradiated with UV light. In particular, Machado et al. [41] encapsulated trans-resveratrol in niosomes, decreasing the percentage of isomerization of trans-resveratrol to cis-resveratrol after 15 min of irradiation with high-energy UV light from 42% to 13%. Similarly, Koga et al. [42] encapsulated resveratrol in sodium caseinate microparticles, also increasing the stability of resveratrol against isomerization by UV light at 356 nm for 1 h; however, the results obtained by these researchers are hardly comparable with those obtained in the present study, since the methodology used to test the protective effects of the materials prepared and the materials prepared themselves were very different. In any case, to the best of our knowledge, no packaging material has ever been prepared in order to study its protective effect on resveratrol against environmental light radiation. 

#### 3.3.2. Film Application as Active Pouches to Pack Extra Virgin Olive Oil

**Figure 4:**
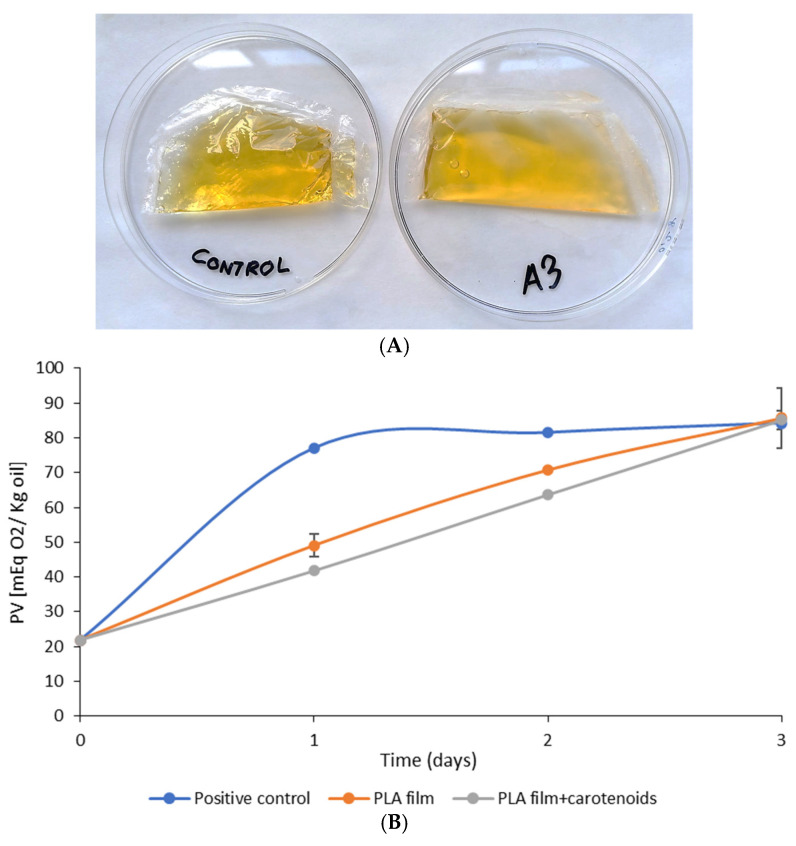
(**A**) EVOO packaged in bags of plain PLA film (**left**) and PLA with egg yolk oil (**right**). (**B**) Peroxide value (PV) changes in free olive oil (blue), olive oil in the PLA film (orange) and olive oil in the A3 PLA film (0.3 g egg yolk oil/g PLA) during the 3-day test.

Taking into account that, as could be seen with the resveratrol, the PLA films with egg yolk oil have a barrier effect against UV/Vis radiation and, as was observed with the DPPH assay, they also possess antioxidant properties, they could be of interest as biodegradable primary packaging intended to protect olive oil against the lipid oxidation that causes its rancidity (Figure 4A).

Figure 4B shows the change in the PV of free olive oil (positive control) and of olive oil packaged with a normal PLA film and with a PLA film loaded with egg yolk oil (film A3), subjected to photo-oxidizing conditions. PV is one of the most widely used parameters to study the quality and deterioration of lipids and fats; it is a biomarker that can indicate the oxidation state of lipids, since it quantifies the presence in oil of hydroperoxides, which are undesirable compounds that result from the primary oxidation of lipids. It can be observed that the free oil (positive control) underwent rapid oxidation, reaching maximum oxidation during the first 24 h of testing, whereas the olive oil contained in the PLA films gradually oxidizes until it reaches the same maximum values as the positive control after 72 h. This difference between the positive control and the olive oil protected by the PLA films is probably due to the film protecting the oil from contact with atmospheric oxygen and from light radiation, thus preventing the formation of reactive singlet oxygen when solar radiation interacts with atmospheric oxygen in the presence of photosensitizers such as chlorophyll and other pigments present in olive oil [43]. However, according to the results shown in Figure 4B, the presence of egg yolk oil in the PLA films gave them an additional protective effect. This is probably due to the release of the carotenoids retained in the film, which have an antioxidant effect and barrier properties against the passage of environmental light. As previously mentioned, the carotenoids will prevent this oil autoxidation or photo-oxidation, capturing free radicals or acting as quenchers, and deactivating the excited singlet oxygens resulting from solar radiation [44,45].

## 4. Conclusions

For the first time, bioactive PLA food packaging materials with egg yolk oil were developed and characterised. The egg yolk oil behaved as a plasticizer, increasing the flexibility of the PLA films and allowing them to be manipulated to prepare food packaging materials. In addition, the egg yolk oil provided the films with antioxidant and light barrier properties, which were tested using photosensitive compounds, such as resveratrol and extra virgin olive oil. The results obtained prove that the PLA films plasticized with egg yolk oil give a statistically significant additional protective effect against environmental light radiation to these sensitive compounds, although this effect may be considered slight. If these films were tailored for a specific application and a higher antioxidant or light barrier capacity were required, these properties could be enhanced by increasing the thickness of the prepared films or by using carotenoids obtained from a vegetable source to increase the concentration of carotenoids in the egg yolk oil. Taking all this into consideration, these materials could provide a good alternative for packing food or easily oxidisable compounds, like cheese, meat or some types of drinks with photosensitive molecules; however, further investigation is required to establish the performance of these materials during a long period of storage time.

## Figures and Tables

**Table 1:** Transmittance and transparency values of PLA films with egg yolk oil, A1 (0.1 g oil/g PLA), A2 (0.2 g/g) and A3 (0.3 g/g), and control PLA film (C).

Films	Transmittance (%)							Transparency
	200 nm	280 nm	350 nm	400 nm	500 nm	600 nm	700 nm	
C	1.29 ± 0.12	84.60 ± 0.42	87.75 ± 0.78	88.60 ± 0.71	89.50 ± 0.85	90.10 ± 0.85	90.55 ± 0.92	1.118 ± 0.060 ^c^
A1	0.04 ± 0.00	47.40 ± 1.98	62.80 ± 3.96	67.85 ± 4.03	73.30 ± 4.67	78.85 ± 3.89	82.00 ± 3.11	1.629 ± 0.323 ^bc^
A2	0.03 ± 0.01	42.65 ± 7.35	62.15 ± 4.60	67.45 ± 3.89	72.75 ± 3.32	77.30 ± 2.83	79.60 ± 2.55	2.113 ± 0.006 ^b^
A3	0.03 ± 0.00	41.10 ± 4.67	58.25 ± 2.19	63.10 ± 1.56	68.30 ± 0.85	72.85 ± 0.21	75.15 ± 0.07	3.368 ± 0.399 ^a^

Different letters in the same column indicate significant differences (*p* < 0.05).

**Table 2:** Colour attributes of PLA films with egg yolk oil, A1 (0.3 g oil/g PLA), A2 (0.2 g/g PLA) and A3 (0.3 g/g PLA), and control PLA film (C).

Film	L*	a*	b*	∆E*	WI	Chroma
C	94.85 ± 1.50 ^a^	0.00 ± 0.42 ^ab^	1.10 ± 0.42 ^c^	-	94.70 ± 1.22 ^a^	1.14 ± 0.41 ^b^
A1	96.60 ± 1.56 ^a^	−0.70 ± 0.42 ^b^	2.40 ± 0.99 ^bc^	2.30 ± 0.48 ^b^	95.59 ± 0.59 ^a^	2.51 ± 1.07 ^b^
A2	96.35 ± 0.21 ^a^	−0.30 ± 0.99 ^ab^	4.40 ± 1.13 ^b^	3.78 ± 0.22 ^ab^	94.20 ± 0.78 ^a^	4.46 ± 1.18 ^b^
A3	94.90 ± 0.87 ^a^	1.05 ± 0.49 ^a^	8.25 ± 1.77 ^a^	7.24 ± 2.18 ^a^	90.24 ± 1.99 ^b^	8.32 ± 1.82 ^a^

Different letters in the same column indicate significant differences (*p* < 0.05).

**Table 3:** Thickness, puncture strength (PS), puncture deformation (PD) and water vapour permeability (WVP) of PLA films with different concentrations of egg yolk oil: A1 (0.1 g/g PLA), A2 (0.2 g/g PLA) and A3 (0.3 g/g PLA). Control (C) is a normal PLA film.

Film	Thickness (mm)	PS (N/mm)	PD (%)	WVP (g × mm/m^2^ × h × kPa)
Control	0.037 ± 0.003 ^b^	407.66 ± 65.94 ^a^	14.69 ± 6.53 ^b^	0.145 ± 0.063 ^ab^
A1	0.040 ± 0.007 ^ab^	315.03 ± 26.82 ^b^	25.20 ± 5.36 ^a^	0.080 ± 0.013 ^b^
A2	0.047 ± 0.008 ^ab^	251.81 ± 32.64 ^b^	21.33 ± 5.66 ^ab^	0.124 ± 0.020 ^ab^
A3	0.050 ± 0.010 ^a^	161.76 ± 18.01 ^c^	14.41 ± 1.97 ^b^	0.177 ± 0.051 ^a^

Different letters in the same column indicate significant differences (*p* < 0.05).

**Table 4:** Protection of PLA films with egg yolk oil against resveratrol isomerization under photooxidant conditions.

	*trans*-Resveratrol (%)	*cis*-Resveratrol (%)
Solution t = 0	100.00 ± 0.00	0.00 ± 0.00
Control 24 h	11.75 ± 0.04	88.25 ± 0.04
Film PLA 24 h	14.71 ± 0.40	85.29 ± 0.40
Film A3 24 h	22.30 ± 0.82	77.70 ± 0.82
Control 48 h	4.84 ± 0.02	95.16 ± 0.02
Film PLA 48 h	13.41 ± 0.22	86.59 ± 0.22
Film A3 48 h	16.43 ± 0.07	83.57 ± 0.07

## Data Availability

Data is contained within the article.
